# Age of Laying Hens Significantly Influences the Content of Nutritionally Vital Lipophilic Compounds in Eggs

**DOI:** 10.3390/foods10010022

**Published:** 2020-12-23

**Authors:** Eun-Young Ko, Ramesh Kumar Saini, Young-Soo Keum, Byoung-Ki An

**Affiliations:** 1 Department of Food Science and Biotechnology of Animal Resources, Konkuk University, Seoul 143-701, Korea; 2 Department of Crop Science, Konkuk University, Seoul 143-701, Korea; saini1997@konkuk.ac.kr (R.K.S.); rational@konkuk.ac.kr (Y.-S.K.); 3 Animal Resources Research Center, Konkuk University, Seoul 143-701, Korea; abk7227@daum.net

**Keywords:** carotenoids, sterol, tocopherols, cholesterol, docosahexaenoic acid (DHA), polyunsaturated fatty acids (PUFAs)

## Abstract

This study aimed to comparatively analyze the carotenoids, tocopherols, fatty acids, and sterols (e.g., cholesterol) in the eggs of laying hens of early (24 weeks), intermediate (42 weeks), and late age (74 weeks), utilizing high-performance liquid chromatography (HPLC), gas chromatography (GC), and GC–mass spectrometry (MS). The results revealed the significantly (*p* < 0.05) highest content of nutritionally vital compounds (per g of egg yolk, fresh weight), which included (all-*E*-)-lutein (21.8 µg), (all-*E*-)-zeaxanthin (13.4 µg), α-tocopherol (76.5 µg), oleic acid (C18:1n9c; 83.3 mg), α-linolenic acid (C18:3n3; 0.68 mg), γ-linolenic acid (C18:3n6; 0.47 mg), arachidonic acid (C20:4n6; 8.11 mg), docosahexaenoic acid (DHA; C22:6n3; 2.06 mg), and total monounsaturated fatty acids (MUFAs; 94.7 mg) and n-3 polyunsaturated fatty acids (PUFAs; 2.74 mg) in the eggs of early-age laying hens compared to intermediate and late-age. Surprisingly, cholesterol was not significantly different in the eggs obtained from the different age groups. In contrast, the fat quality indices, including the lowest atherogenic index (AI) and thrombogenic index (TI) values and high hypocholesterolemic/hypercholesterolemic (h/H) fatty acid ratio, indicated the health-beneficial potential associated with fat intake from the eggs of intermediate and late-age laying hens. Overall, the results of the present investigation suggest that eggs from early-age laying hens can be recommended for a higher intake of antioxidant carotenoids and tocopherols, while the eggs from intermediate and late-age hens can be recommended for the intake of good quality fats.

## 1. Introduction

Eggs are a vital component of healthy Mediterranean and vegetarian diets [1] rich in health-beneficial bioactive compounds, including, folate, riboflavin, cobalamin (vitamin B12), tocols (vitamin E; tocopherols and tocotrienols), carotenoids (e.g., lutein and zeaxanthin), and omega-3 (n−3) polyunsaturated fatty acids (PUFAs) [2,3]. Hen eggs are considered an antioxidant food commodity because they contain a substantial amount of redox-regulating proteins (e.g., ovalbumin, ovotransferrin, and phosvitin), tocols, selenium, and carotenoids [4]. Lutein and zeaxanthin are key macular pigments that may protect from age-related macular degeneration, and these pigments are highly bioavailable when present in lipid-rich egg yolks. Thus, egg intake was shown to significantly increase the plasma level of these pigments [5]. Vitamin E (consists of α-, β-, γ-, and δ-tocopherol and α-, β-, γ-, and δ-tocotrienol), collectively referred to as tocochromanols or tocols are well known chain-breaking antioxidants [6]. On average, an egg contains around 1.1 mg of vitamin E, which is equivalent to 8.5% of the recommended daily allowance (RDA) [4]. 

The intense golden yellow pigmentation of the yolk, low levels of cholesterol, and higher n-3 PUFA content determine the commercial value (consumer acceptance) of eggs [7,8,9]. An adequate and balanced intake of n−3 PUFAs (ideally in a 1:1 ratio with n−6 PUFAs) is beneficial for the protection against chronic and metabolic diseases [6,10]. While a low intake of cholesterol is recommended by adopting a healthy dietary pattern (e.g., Mediterranean-style diet) [11]. The composition of cholesterol and n−3 PUFAs in eggs mainly depends upon the type of the avian species [12,13]. However, to a great extent, it is also substantially influenced by genotype (breed, line, hybrid), the housing systems, age, and feeding [2,3,9,14]. Tang et al. [15] observed a significant increase in α-tocopherol content with the age (24, 28, 32, and 36 weeks) of laying hens with nearly constant γ-tocopherol contents.

To the best of our knowledge, the detailed reports on how nutritionally vital lipophilic compounds in eggs are influenced by the age of laying hens are not available. Thus, this study aimed to investigate the impact of the age of laying hens on the key bioactive lipophilic compounds including carotenoids, tocopherols, fatty acids, and sterols. The results obtained herein are anticipated to contribute significantly to demonstrating the comparative nutritional significance of eggs obtained from early, intermediate, and late-age laying hens. 

## 2. Materials and Methods 

### 2.1. Reagents and Standards

Authentic (all-*E*)-lutein, (all-*E*)-zeaxanthin standards were purchased from Cayman Chemical Company, Ann Arbor, MI, USA. The fatty acid standard mixture (37 Component FAME Mix, CRM47885), tocols (mixture of α-, β-, γ-and δ-tocopherol, and α-, β-, γ-, and δ-tocotrienol), and cholesterol were obtained from Merck Ltd., Seoul, South Korea. All organic solvents used for the extractions were of HPLC grade and obtained from Daejung Chemicals and Metals Co., Ltd., Siheung-si, Korea.

### 2.2. Animals, Diets, Sampling, and Sample Preparation

For the experimental purpose, a total of 90 Lohmann Lite Brown laying hens of early (24 weeks, *n* = 30), intermediate (42 weeks, *n* = 30), and late age (74 weeks, *n* = 30) were maintained at a commercial farm located in Yeoncheon-gun, Gyeonggi-do, South Korea. Laying hens of these age groups are used for the commercial production of eggs. The experimental protocol was approved by the institutional animal care and use committee at Konkuk University (KU20169). The main ingredients and the nutrient composition of the experimental diets are shown in Table 1. All eggs were laid in the same period from different laying hens in August 2020. The egg samples (*n* = 30) obtained from each age group were brought to the laboratory, divided into six subgroups (five eggs in each subgroup), the egg yolk was separated from the respective egg white, homogenized using a food blender, and stored at −20 °C until analysis. The eggs obtained from early, intermediate, and late-age laying hens weighed an average of 53.9 ± 0.49, 56.7 ± 0.60, and 56.9 ± 0.66 g, respectively. Similarly, 20.1 ± 0.55, 24.1 ± 1.14, and 25.1 ± 0.78% egg yolk (% in weight) was recorded in the eggs obtained from early, intermediate, and late-age laying hens, respectively.

A fraction of (15 g) the egg yolk samples from the homogenized samples was used for the extraction of major lipophilic compounds. For each group, the analytical parameters were determined in six independent experiments (one determination from each subgroup of five eggs).

### 2.3. Extraction of Major Lipophilic Compounds

**Figure 1:**
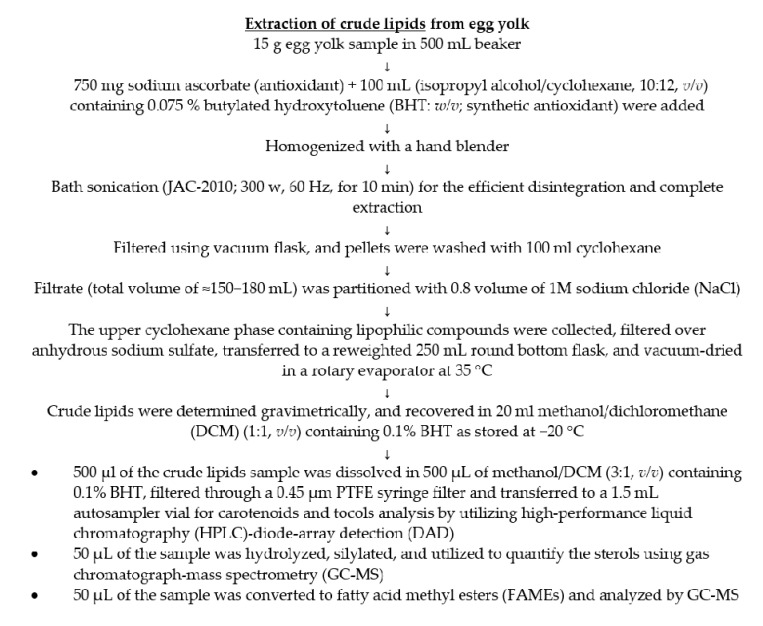
Method for the simultaneous extraction of major lipophilic compounds.

The major lipophilic compounds, including fatty acids, tocols, and sterols, were simultaneously extracted following the previous method [16,17,18,19] with minor modification. The detailed procedure is illustrated in Figure 1. The extracted crude lipids were aliquoted into three fractions, as illustrated in Figure 1, and utilized accordingly. Tocols and carotenoids were analyzed before hydrolysis as hydrolysis may cause significant degradation of carotenoids and tocopherol [16,20]. Moreover, in the preliminary studies, no esterified carotenoids were detected in the egg yolk and, thus, hydrolysis was avoided. However, for the analysis of sterols, crude lipids were hydrolyzed before GC–MS analysis [16]. 

### 2.4. HPLC-DAD Analysis of Carotenoids and Tocols

Tocols and carotenoids were analyzed using an Agilent HPLC system (Model 1100; Agilent Technologies Canada, Inc., Mississauga, ON, Canada) equipped with a DAD, autosampler, dual pump, and a YMC C30 column (250 × 4.6 mm^2^, 5 μm; YMC, Wilmington, NC, USA). The solvent system was composed of methanol/water (95:5; mobile phase A) and methyl tertiary butyl ether (MTBE)/methanol/water (90:7:3; mobile phase B). A gradient elution program was followed (0–100% B) for a total of 45 min of analysis time with a 5-min post-run at 0% B, at a flow rate of 1 mL/min. Samples were scanned (180–800 nm) with a 0.05 min (1 s) response time at detection wavelengths of 295 nm (for tocols) and 450 nm (for carotenoids). The bandwidth and reference wavelengths were used as previously optimized [21].

### 2.5. Analysis of Sterols and Fatty Acid Methyl Esters (FAMEs)

FAMEs were quantitatively analyzed following our recently optimized method [22] utilizing a gas chromatograph (GC) (Agilent 7890B, Agilent Technologies Canada, Inc.) equipped with a flame ionization detector (FID), autoinjector, and an SP-2560 capillary GC column (100 m, 0.20 μm film thickness, 0.25mm ID; Merck KGaA, Darmstadt, Germany). The injector and detectors were precisely maintained at 250 and 260 °C, respectively. The inlet flow was 2 mL/min with a constant pressure of 54 psi. The FID parameters of airflow, hydrogen (H_2_) fuel flow, and make up flow (nitrogen, N_2_) were set to 400, 30, and 25 mL/min, respectively. The column oven temperature was kept at 140 °C for 5 min, then progressively increased to 240 °C over 25 min (4 °C/min of linear temperature program), and held at 240 °C for 15 min. The column was equilibrated by a 5-min post-run at 140 °C. The FAMEs were identified by comparing them to the retention times of authentic standards. Additionally, for more precise qualitative analysis, the mass spectra were recorded using a GC–MS system (QP2010 SE; Shimadzu, Japan), following the optimized thermal GC-FID analysis program. The identity of the FAMEs was confirmed by comparing their fragmentation pattern with authentic standards. 

**Figure 2:**
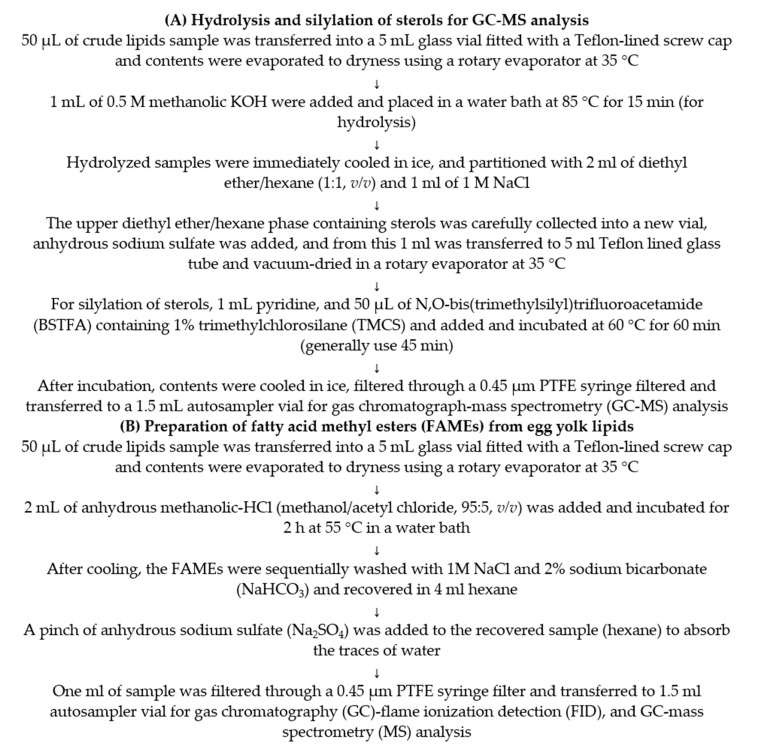
(**A**) Method for hydrolysis of crude lipid extract for sterol analysis and (**B**) the preparation of fatty acid methyl esters (FAMEs).

Sterols were analyzed after silylation as illustrated in Figure 2A, utilizing QP2010 SE GC–MS equipped with an autoinjector and a fused silica Rxi-5ms column (30 m, 0.5 μm film thickness, 0.25 mm ID; Restek Corporation, Bellefonte, PA, USA). Helium was used as a carrier gas, maintained at a linear flow of 36.7 cm/min (5.8 mL/min total flow). The injector and MS ion source were precisely maintained at 260 °C, while the MS interface was maintained at 280 °C. The column oven temperature was kept at 200 °C for 1 min, then progressively increased to 300 °C with a linear increase of 3 °C/min, and held at 300 °C for 26 min. Samples and standards (1 µL) were injected in a 1:2 split ratio. The sterols were identified by comparing their retention time and fragmentation pattern to those of authentic standards. 

### 2.6. Calculation of Fat Quality Indices

The fatty acid profile was used to determine several nutritional parameters of the egg yolk lipids, including the ratios of hypocholesterolemic (h)/hypercholesterolemic (H) fatty acids, PUFAs/monounsaturated fatty acids (MUFAs), PUFAs/saturated fatty acids (SFAs), and n-6 PUFAs/n-3 PUFAs [19,23,24]. Additionally, thrombogenic index (TI) [25] and atherogenic index (AI) [23] were calculated as the following equations:

(1) $$h/H=\frac{C18:1n9c\;+\;C18:2n6c\;+\;C18:3n3c\;+\;C18:3n6c\;+\;C20:2n6\;+\;C20:3n6\;+\;C20:4n6\;+\;C22:6n3}{C14:0\;+\;C16:0}$$

(2) $$\mathrm{TI}=\frac{C14:0\;+\;C16:0\;+\;C18:0}{\left(0.5\;\times \;MUFAs)\;+\;(0.5\;\times \;n6\;FUFAs)\;+\;(3\;\times \;n3\;FUFAs)\;+\;(\frac{n3\;FUFAs}{n6\;FUFAs}\right)}$$

(3) $$\mathrm{AI}=\frac{\left(4\;\times \;C14:0\right)+C16:0}{MUFAs+FUFAs}$$

### 2.7. Statistical Analysis and Quality Control

A total of six independent experiments were performed in samples from each age group. The values from all six independent experiments were averaged and presented as means ± standard deviation. The data were analyzed by one-way analysis of variance (ANOVA) and homogenous subsets were determined using Tukey’s honestly significant difference (HSD) test with a significance level of *p* < 0.05 utilizing IBM Statistics 25.0 software (IBM, Armonk, NY, USA; version 25). 

Quality control data were not described in the present investigation, as fatty acids and other lipophilic compounds are extracted and quantified according to previously optimized methods. Moreover, the GC-FID method used for the quantification of fatty acids was recently validated [22].

## 3. Results and Discussion

### 3.1. Carotenoid Composition

**Figure 3:**
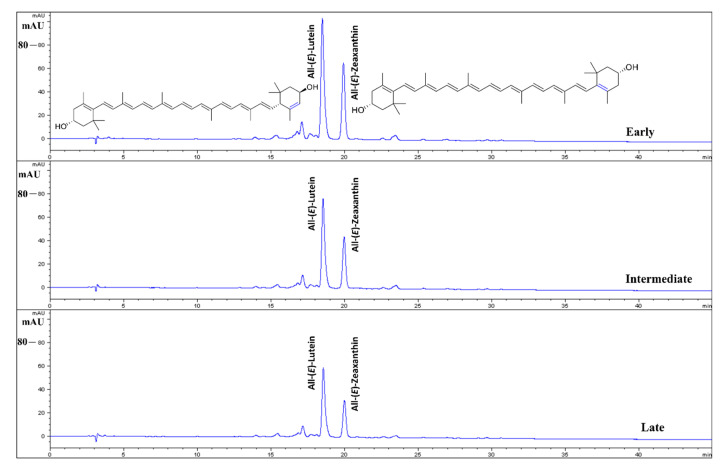
High-performance liquid chromatography (HPLC)-diode-array detection (DAD) chromatograms (450 nm) showing the dominance of (all-*E*)-lutein (RT—18.517 min) and (all-*E*)-zeaxanthin (RT—19.955 min) in the egg yolks of eggs obtained from early, intermediate, and late-age laying hens. All the chromatograms are on the same scale. RT: retention time.

**Figure 4:**
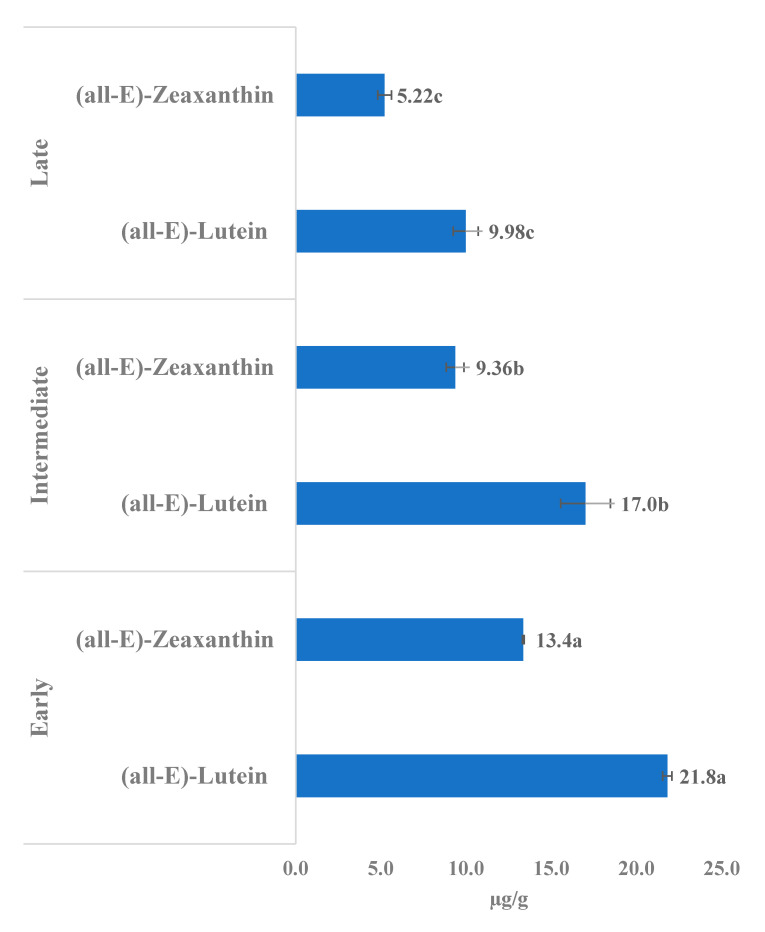
Content (µg/g egg yolk; fresh weight) of (all-*E*)-lutein and (all-*E*)-zeaxanthin in the egg yolks of eggs from early, intermediate, and late-age laying hens. The values are the mean ± standard deviation of six independent experiments. Different letters (a–c; in data labels) represent significant differences (*p* < 0.05) between early, intermediate, and late-age laying hens.

The HPLC-DAD analysis of carotenoids in the egg yolks revealed the dominance of (all-*E*)-lutein (β,ε-carotene-3,3′-diol) and (all-*E*)-zeaxanthin (β,β-carotene-3,3′-diol); (Figure 3). In the comparison, the eggs yolks obtained from early-age laying hens showed the significantly highest (*p* < 0.05) content of (all-*E*)-lutein (21.8 µg/g Fresh Weight; FW FW) and (all-*E*)-zeaxanthin (13.4 µg/g FW) compared to laying hens of intermediate and late-age (Figure 4). Moreover, contents calculated on a per egg basis also showed the significantly highest (*p* < 0.05) content of (all-*E*)-lutein (235.1 µg/egg) and (all-*E*)-zeaxanthin (144.0 µg/egg) in the egg yolks obtained from early-age laying hens, compared to 229.8 and 126.5 µg of (all-*E*)-lutein and 126.5 and 75.5 µg of (all-*E*)-zeaxanthin in egg yolks obtained from intermediate, and late-age laying hens, respectively.

Lutein and zeaxanthin are the major xanthophyll carotenoids primarily deposited in the human retina and protect the macula from damage by blue light, improve vision, and scavenge harmful radical species [2,26]. The egg yolks of various avian species, including hens, are a rich dietary source of these vital carotenoids [13,27]. The carotenoid content in egg yolks between and within species is significantly different, with a large dependence upon feeding habits [2,13,27]. Perry et al. [28] recorded 7.87 µg/g (all-*E*)-lutein and 7.62 µg/g (all-*E*)-zeaxanthin in the egg yolks of eggs obtained from commercial eggs marketed in the United States. In a comparison with Perry et al. [28], in the present investigation, we recorded the higher amount of (all-*E*)-lutein (9.98–21.8 µg/g) and low-to-higher (all-*E*)-zeaxanthin (5.22–13.4 µg/g) in the egg yolks of eggs obtained from laying hens of different age groups. Moreover, in the study of Perry et al. [28], (all-E)-lutein contents (7.87 µg/g) were almost similar to the (all-*E*)-zeaxanthin (7.62 µg/g), while, in the present investigation, within each age group, (all-E)-lutein contents were nearly 50% higher than (all-*E*)-zeaxanthin, for instance, 21.8 µg/g FW of (all-*E*)-lutein and 13.4 µg/g FW (all-*E*)-zeaxanthin in the egg yolk of early-age laying hens. The same was visible in the carotenoid chromatograms (Figure 3). The occurrence of higher levels of lutein compared to zeaxanthin in egg yolk is reported in other studies also [27]. Brulc et al. [27] recorded significant variations in the content of lutein and zeaxanthin in egg yolks obtained from husbandry classifications of ecological, free-range, barn, and caged hens of 8.1, 26.9, 29.7, and 37.2 µg/g lutein and 5.4, 12.4, 9.8, and 11.2 µg/g of zeaxanthin, respectively. The zeaxanthin contents of 11.2 µg/g recorded from egg yolks cage-reared hens are in agreement with the zeaxanthin contents in the present study (early-age, cage-reared). The contents of lutein and zeaxanthin in the egg yolk can be substantially enhanced by the dietary supplementation of carotenoids in the feed [26]. For instance, 46.0 µg/g (all-*E*)-lutein and 24.3 (all-*E*)-zeaxanthin were found [26] in the eggs produced by laying hens fed natural lutein and zeaxanthin (<80 ppm).

Cornmeal is the key source of lutein and zeaxanthin in chicken feed [2]. In the present study, 54.8–57.3% corn seeds, 12.0–13.2% corn distiller’s corn, and 1–4.2% corn gluten meal were added to the feed of early, intermediate, and late-age laying hens, which showed no substantial difference in cornmeal composition (total cornmeal, distiller’s corn, and corn gluten meal) between the various classes. The high content of lutein and zeaxanthin in the egg yolks of eggs obtained from early-age laying hens is probably the result of the active metabolism of early-age laying hens. Anderson [29] recorded the highest content of vitamin A (3.72 IU/50 g) at 50 weeks, compared to 62 weeks (2.42 IU/g), and 74 weeks. The highest content of lutein and zeaxanthin recorded in the eggs of early-age laying hens suggest that these eggs can be used for the formulation of a lutein and zeaxanthin-rich diet.

### 3.2. Sterol and Tocol Composition

**Figure 5:**
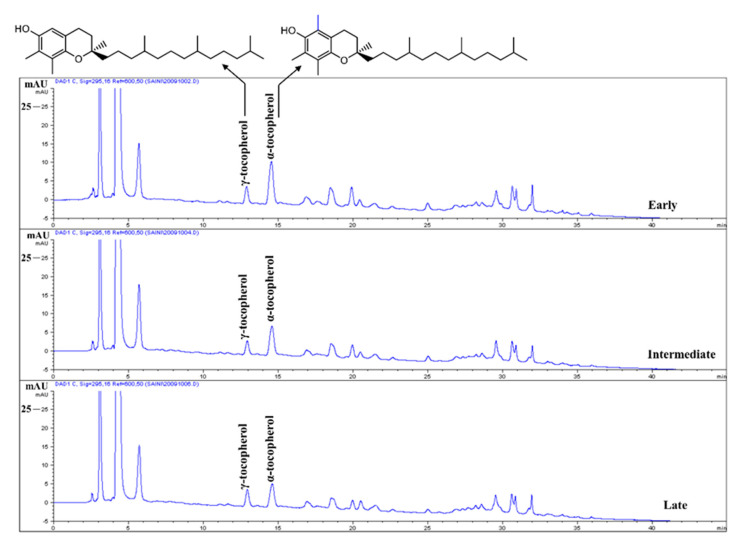
High-performance liquid chromatography (HPLC)-diode-array detection (DAD) chromatograms (295 nm) of γ-tocopherol (RT—12.925 min) and α-tocopherol (RT—14.587 min) from the egg yolks of eggs obtained from early, intermediate, and late-age laying hens. All the chromatograms are on the same scale. RT: retention time.

**Figure 6:**
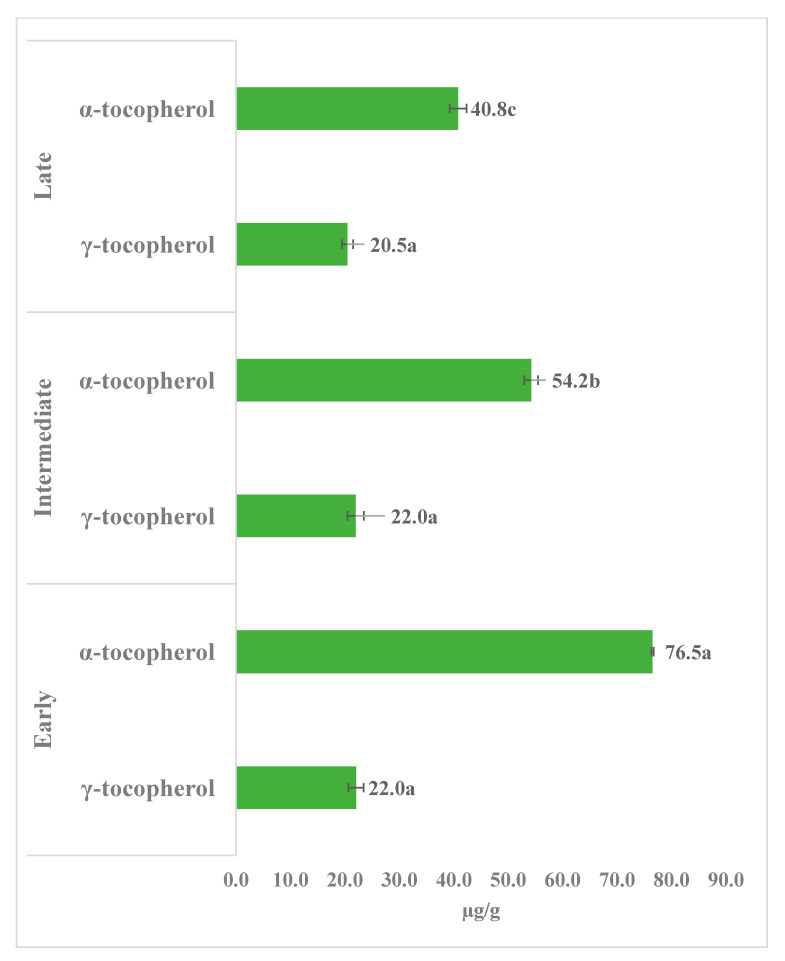
Content (µg/g egg yolk; fresh weight) of γ-tocopherol and α-tocopherol in the yolks from early, intermediate, and late-age laying hens. The values are the mean ± standard deviation of six independent experiments. Different letters (in data labels) represent significant differences (*p* < 0.05) between early, intermediate, and late-age laying hens.

Tocols are composed of α-, β-, γ-, and δ-tocotrienols and α-, β-, γ-, and δ-tocopherols, determined by the numbers and position of methyl (−CH_3_) groups present at the 5- and 7-positions of the chromanol ring [6]. In the present study, γ-tocopherol and α-tocopherol were identified as the major tocols in egg yolk by HPLC-DAD analysis (Figure 5). In the comparison, the yolk of eggs obtained from early-age (24 weeks) laying hens showed the significantly highest (*p* < 0.05) amount of α-tocopherol (76.5 µg/g FW) and γ-tocopherol (22.0 µg/g FW) compared to laying hens of early and intermediate age (Figure 5). Moreover, the total contents calculated on a per egg basis also showed the significantly highest (*p* < 0.05) contents of α-tocopherol (825.0 µg/egg) and total tocopherols (1062.9 µg/egg; the sum of γ-tocopherol and α-tocopherol) in the egg yolks obtained from early-age laying hens, compared to 733.0 and 1030.0, and 590.0 and 886.1 µg/egg of α-tocopherol and total tocopherols in egg yolks obtained from intermediate and late-age laying hens, respectively. Interestingly, although the contents of γ-tocopherol were similar (20.5–22.0 µg/g FW; nonsignificant at *p* < 0.05) among yolks obtained from early-, intermediate-, and late-age laying hens (Figure 6), the total contents of γ-tocopherol was significantly highest (296.1–297.0 µg/egg; nonsignificant at *p* < 0.05) in the egg obtained from intermediate and late-age laying hens owing to the higher total amount of egg yolk (13.5 and 14.5 g, respectively), compared to early-age laying hens (10.8 g).

Consumption of one egg (from early-age laying hens) can supply 7.09% of the daily requirement of vitamin E for adult men and nonlactating women (19 years and older), considering the recommended dietary allowance (RDA) of 15 mg/day [6,30], and 1.06 mg/egg of total tocopherols (vitamin E) in the egg obtained from the early-age laying hens. These findings are in agreement with the previous report [4].

The α-tocopherol content found in the eggs of early-age (24 weeks) laying hens in the present study was lower than previously reported, whereas the γ-tocopherol content was higher [15]. Tang et al. [15] recorded 92.9 µg/g α-tocopherol and 11.0 µg/g γ-tocopherol from egg yolks obtained from 24-week-old laying hens. Interestingly, Tang et al. [15] observed a significant increase in α-tocopherol content with the age (24, 28, 32, and 36 weeks) of laying hens with nearly constant γ-tocopherol content. In the present study, we also observed similar γ-tocopherol content with the age (24, 42, and 74 weeks) of the laying hens. However, in the present study, the α-tocopherol content decreased with the age of the laying hens (Figure 6). The exact reason for these differences is unknown. However similar to the (all-*E*)-lutein and (all-*E*)-zeaxanthin, the higher levels of α-tocopherol recorded in the egg yolks of eggs of early-age laying hens are probably the result of the active metabolism of early-age laying hens.

**Figure 7:**
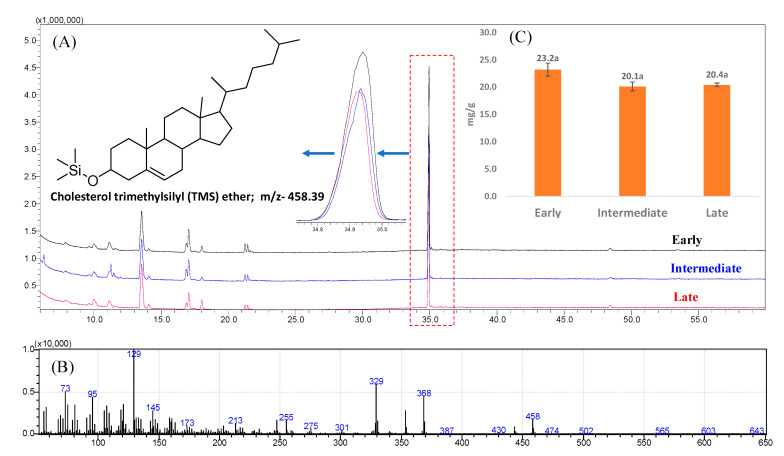
(**A**) The gas chromatography–mass spectrometry (GC–MS) total ion chromatogram (TIC) of cholesterol (retention time: 34.934 min). (**B**) The GC–mass spectrum of cholesterol from egg yolk. (**C**) Content (mg/g egg yolk; fresh weight) of cholesterol in the yolks from early, intermediate, and late-age laying hens. The values are the mean ± standard deviation of six independent experiments. The same letters (a; in data labels) represent differences is not significant (*p* > 0.05) between early, intermediate, and late-age laying hens.

In the present study, cholesterol was the single most-dominant sterol in egg yolk (Figure 7). Surprisingly, cholesterol, which is generally considered a risk factor for developing cardiovascular disease (CVD) [31], and known for initiation of pathophysiological angiogenesis [32], was not significantly different in the eggs obtained from the laying hens of different age groups (23.2, 20.1, and 20.4 mg/g FW in eggs yolks of early, intermediate, and late-age chickens, respectively). These cholesterol values can be converted to 250.3, 271.8, and 295.1 mg/egg considering egg yolk weights of 10.8, 13.5, and 14.5 g from the eggs of early, intermediate, and late-age chickens used in the present study. Attia et al. [9] reported 14.3–15.9 mg/g FW of cholesterol in chicken eggs marketed in Jeddah City, Saudi Arabia. In contrast, 4.9–7.4 mg/g FW cholesterol was reported in the egg yolks obtained from different genotypes of laying hens at 50 weeks of age [14]. 

Although earlier evidence suggested that a certain level of cholesterol intake can be harmful [33], more recent findings have suggested adopting a healthy dietary pattern with the balance of a whole diet outweighs the impact of a single nutrient, i.e., cholesterol level in eggs [1,11]. Moreover, a recent meta-analysis study comprising 23 prospective studies suggested that higher consumption of eggs (more than 1 egg/day) was not associated with an increased risk of cardiovascular disease, but was associated with a significant reduction in the risk of coronary artery disease [34]. Additionally, the results of previous studies and the present study indicate that the consumption of 1 egg/day may provide < 300 mg cholesterol, which is within the safe limits.

### 3.3. Fatty Acid Composition and Fat Quality Indices

**Figure 8:**
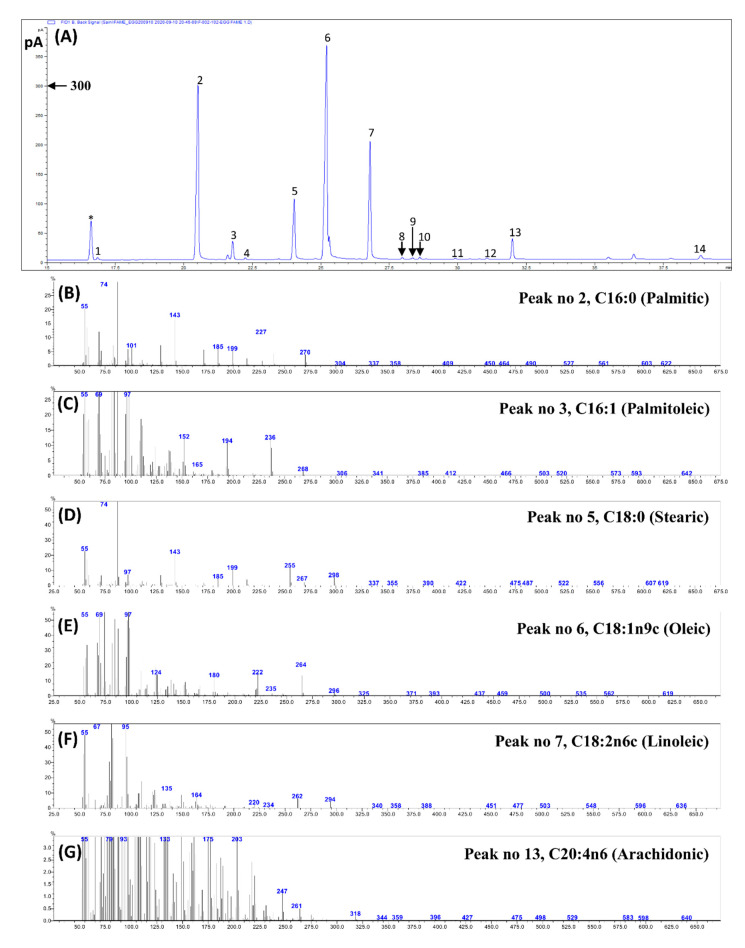
(**A**) Representative gas chromatography (GC)-flame ionization detection (FID) profiles of fatty acid methyl esters (FAMEs) from egg yolks. The peak numbers correspond to those used in Table 2. (**B–G**) The GC–mass spectrum of major FAMEs identified. The retention time of each identified FAMEs is given in Table 2.

In the present study, 14 fatty acids were identified and quantified utilizing GC-FID and GC–MS analyses (Table 2). A representative GC-FID chromatogram of the fatty acids identified and the GC–mass spectra of the major fatty acids identified are shown in (Figure 8). In all the studied egg yolk samples, oleic acid (C18:1n9c) was found in the highest concentration (88.3–71.5 µg/g FW), accounting for 37.4–37.2% of the total fatty acids, followed by palmitic (C16:0; 64.62–51.3 µg/g FW), linoleic (C18:2n6c; 40.7–37.8 µg/g FW), arachidonic (C20:4n6; 8.11–6.32 µg/g FW), and palmitoleic acid (C16:1; 6.00–4.01 µg/g FW). These five fatty acids together accounted for 88.3% (late age) to 87.5% (intermediate age) of the total fatty acids. The predominance of oleic, palmitic, and linoleic acid in hen eggs is consistent with the results of previous studies [7,9,12,15]. Among the eggs obtained from early, intermediate, and late-age chickens, the highest content (mg/g egg yolk; FW) of health-beneficial fatty acids, including oleic acid (C18:1n9c; 83.3), α-linolenic acid (C18:3n3; 0.68), γ-linolenic acid (C18:3n6; 0.47), arachidonic acid (C20:4n6; 8.11), docosahexaenoic acid (DHA; C22:6n3; 2.06), and total monounsaturated fatty acids (MUFAs; 94.7) were found in the eggs of early-age laying hens, suggesting greater health benefits from eggs obtained from early-age hens. The significantly highest total contents of n-3 polyunsaturated fatty acids (PUFAs; 2.74 mg/g FW) were recorded from the eggs of early-age laying hens. However, with the highest MUFAs and n-3 PUFAs, the significantly highest total contents of saturated fatty acids (SFAs; 88.5 mg/g FW) were also recorded from the eggs of early-age laying hens.

Surprisingly, in the present investigation, despite the higher (235.9 mg/g FW; the sum of SFAs, MUFAs, and PUFAs) contents of total fatty acids were recorded in the egg yolk of early-age laying hens, compared to the intermediate (199.6 mg/g FW) and late-age laying hens (198.6 mg/g FW), the % crude lipids were recorded similar (34.8–36.2%, nonsignificant at *p* < 0.05; table 2) among egg yolk of different age groups. These observations suggest that other lipophilic components are probably enriched in the egg yolk of intermediate and late-age laying hens.

In view of the risk of cardiovascular (CVD) and other chronic diseases associated with the consumption of SFAs [10], fats with PUFA/SFA ratios of greater than 0.45 are considered safe for human consumption [24]. In the present study, the PUFA/SFA ratios ranged from 0.71 (intermediate egg) to 0.60 (early eggs) (Table 3), showing that egg fat (irrespective of the age of the laying hen) fell within the recommendations. Moreover, the fats with lower AI and TI, and higher ratios of h/H fatty acids are recommended for minimizing the risk of CVD [23]. Among the fat quality (nutritional) indices, AI and TI are commonly used to assess the composition of fatty acids of seaweeds, crops, meat, fish, dairy products as they outline significant implications and provide clear evidence of nutritional quality [35]. In the present study, a minor but significant difference was recorded for AI and TI values, and h/H fatty acid ratios of egg lipids obtained from the eggs of early, intermediate, and late-age laying hens. The significantly lowest AI values (0.42–0.43) and TI values (1.04–1.05), and highest h/H fatty acid ratios (3.30–2.37) were obtained from the eggs of intermediate and late-age laying hens, which did not differ significantly from each other. These observations indicate the health-beneficial potential associated with fat intake from eggs of intermediate and late-age chickens.

## 4. Conclusions

The present study revealed the highest content of vital nutrients, including (all-*E*)-lutein, (all-*E*)-zeaxanthin, α-tocopherol, and n-3-PUFAs in the eggs of early-age laying hens, compared to intermediate and late-age laying hens. Moreover, the total contents calculated on a per egg basis showed the significantly highest content of (all-*E*)-lutein, (all-*E*)-zeaxanthin, α-tocopherol, and total tocopherols in the egg yolks of early-age laying hens, compared to intermediate-, and late-age laying hens. Consumption of one egg (from early-age laying hens) can supply 7.09% of the daily requirement of vitamin E for adult men and nonlactating women. In contrast, the AI and TI, fat quality indices, and high h/H fatty acid ratio indicated the health-beneficial potential associated with fat intake from eggs of intermediate and late-age laying hens. Overall, the results of the present investigation suggest that eggs from early-age laying hens can be recommended for a higher intake of antioxidant carotenoids and tocopherols, whereas the eggs from intermediate and late-age hens can be recommended for the intake of good quality fats.

## Figures and Tables

**Table 1:** The composition (%, *w*/*w*) and calculated analysis of the basal diet.

Ingredients	Age Groups		
	Early (24 wk)	Intermediate (42 wk)	Late (74 wk)
Corn Soybean meal Canola meal Corn gluten meal Distiller’s corn Tallow	54.805 11.000 5.514 4.150 12.000 1.100	56.031 10.500 5.301 2.460 13.000 1.050	57.281 10.000 5.675 1.000 13.166 1.000
Layer vitamin premix Lysine, 55% Monocalcium phosphate (MCP) Methionine (Met) 90% Limestone Choline-chloride 50% Salt Layer Mineral Mix Phytase	0.110 0.256 0.636 0.040 9.914 0.005 0.250 0.170 0.050	0.105 0.245 0.590 0.058 10.200 - 0.250 0.160 0.050	0.100 0.222 0.542 0.073 10.481 - 0.250 0.160 0.050
Total	100	100	100
Calculated analysis			
Crude protein, % Calcium (Ca), % Phosphorus (P), % Available P, % Cysteine + Met, % Metabolizable energy, kcal/kg	17.0 3.9 0.52 0.23 0.69 2800	16.0 4.0 0.51 0.22 0.66 2790	15.0 4.1 0.50 0.21 0.64 2780

**Table 2:** Fatty acid composition of egg yolks obtained from early (24 wk), intermediate (42 wk), and late age laying (74 wk) hens.

Peak No	Fatty Acid Methyl Ester (FAME)	RT (min)	Early (24 wk)	Intermediate (42 wk)	Late(74 wk)
1	C14:0 (Myristic)	16.848	0.74 ± 0.03 ^a^ (0.31)	0.60 ± 0.06 ^b^ (0.30)	0.62 ± 0.02 ^b^ (0.31)
2	C16:0 (Palmitic)	20.508	64.6 ± 3.80 ^a^ (27.4)	51.3 ± 3.24 ^b^ (25.7)	52.3 ± 1.04 ^b^ (26.3)
3	C16:1 (Palmitoleic)	21.774	6.00 ± 0.19 ^a^ (2.55)	4.01 ± 0.60 ^c^ (2.00)	5.06 ± 0.35 ^b^ (2.55)
4	C17:0 (Heptadecanoic)	22.235	0.88 ± 0.09 ^a^ (0.37)	0.89 ± 0.09 ^a^ (0.45)	0.70 ± 0.03 ^b^ (0.35)
5	C18:0 (Stearic)	24.02	22.3 ± 1.41 ^a^ (9.43)	19.8 ± 0.06 ^b^ (9.91)	18.58 ± 0.12 ^b^ (9.35)
6	C18:1n9c (Oleic)	25.204	88.3 ± 5.26 ^a^ (37.4)	71.5 ± 1.49 ^b^ (35.8)	73.8 ± 0.83 ^b^ (37.2)
7	C18:2n6c (Linoleic)	26.789	40.5 ± 0.74 ^a^ (17.2)	40.7 ± 1.95 ^a^ (20.4)	37.8 ± 0.25 ^b^ (19.1)
8	C18:3n6 (γ-Linolenic)	27.964	0.47 ± 0.11 ^a^ (0.20)	0.34 ± 0.01 ^b^ (0.17)	0.27 ± 0.02 ^b^ (0.14)
9	C20:1n9 (cis-11-Eicosenoic)	28.343	0.39 ± 0.05 ^a^ (0.16)	0.32 ± 0.02 ^a^ (0.16)	0.37 ± 0.03 ^a^ (0.18)
10	C18:3n3 (α-Linolenic)	28.606	0.68 ± 0.02 ^a^ (0.29)	0.63 ± 0.05 ^b^ (0.31)	0.54 ± 0.05 ^b^ (0.27)
11	C20:2n6 (cis-11,14-Eicosadienoic)	29.898	0.32 ± 0.02 ^a^ (0.14)	0.34 ± 0.03 ^a^ (0.17)	0.34 ± 0.02 ^a^ (0.17)
12	C20:3n6 (cis-8,11,14-Eicosatrienoic)	31.058	0.51 ± 0.04 ^a^ (0.22)	0.49 ± 0.03 ^a^ (0.25)	0.41 ± 0.02 ^b^ (0.21)
13	C20:4n6 (Arachidonic)	31.985	8.11 ± 0.71 ^a^ (3.44)	7.1 ± 0.25 ^b^ (3.58)	6.32 ± 0.21 ^b^ (3.18)
14	C22:6n3 (cis-4,7,10,13,16,19-Docosahexaenoic)	38.862	2.06 ± 0.18 ^a^ (0.87)	1.6 ± 0.18 ^b^ (0.80)	1.48 ± 0.02 ^b^ (0.75)
	Total SFAs		88.5 ± 5.32 ^a^ (37.5)	72.6 ± 3.36 ^b^ (36.4)	72.16 ± 1.17 ^b^ (36.3)
	Total MUFAs		94.7 ± 5.50 ^a^ (40.1)	75.8 ± 2.11 ^b^ (38.0)	79.27 ± 0.47 ^b^ (39.9)
	Total PUFAs		52.7 ± 1.76 ^a^ (22.4)	51.2 ± 1.65 ^a^ (25.7)	47.20 ± 0.51 ^b^ (23.8)
	Crude lipids (%)		34.7 ± 0.39 ^a^	34.8 ± 0.32 ^a^	36.2 ± 0.29 ^a^

Values (mg/g egg yolk; fresh weight) are the mean ± standard deviation from an average of six independent experiments. In the parentheses, contents are expressed as % of total fatty acids. SFAs: total saturated fatty acids; MUFAs: total monounsaturated fatty acids; PUFAs: total polyunsaturated fatty acids; RT: retention time; wk: weeks. Different letters (^a–c^) within a row represent significant differences (*p* < 0.05). RT: retention time.

**Table 3:** The fat quality indices of lipids in the eggs of early (24 wk), intermediate (42 wk), and late-age laying (74 wk) hens.

	Early (24 wk)	Intermediate (42 wk)	Late (74 wk)
PUFAs: SFAs	0.60 ± 0.02 ^c^	0.71 ± 0.01 ^a^	0.65 ± 0.00 ^b^
PUFAs: MUFAs	0.56 ± 0.01 ^c^	0.68 ± 0.00 ^a^	0.60 ± 0.01 ^b^
n3 PUFA	2.74 ± 0.15 ^a^	2.22 ± 0.13 ^b^	2.03 ± 0.07 ^b^
n6 PUFA	49.9 ± 1.61 ^a^	49.0 ± 1.78 ^a^	45.2 ± 0.45 ^b^
n6/n3	18.2 ± 0.42 ^c^	22.2 ± 2.10 ^a^	22.3 ± 0.52 ^a^
h/H	2.16 ± 0.02 ^b^	2.37 ± 0.09 ^a^	2.30 ± 0.05 ^a^
AI	0.46 ± 0.00 ^a^	0.42 ± 0.01 ^b^	0.43 ± 0.01 ^b^
TI	1.09 ± 0.01 ^a^	1.04 ± 0.02 ^b^	1.05 ± 0.01 ^b^

Values are the mean ± standard deviation from an average of six independent experiments. SFAs: total saturated fatty acids; MUFAs: total monounsaturated fatty acids; PUFAs: total polyunsaturated fatty acids; h/H: ratio of hypocholesterolemic (h)/hypercholesterolemic (H) fatty acids; AI: atherogenic index; TI: thrombogenic index; wk: weeks. Different letters (^a–c^) within a row represent significant differences (*p* < 0.05).

## Data Availability

Data is contained within the article.
